# The expression of SIRT1 in articular cartilage of patients with knee osteoarthritis and its correlation with disease severity

**DOI:** 10.1186/s13018-016-0477-8

**Published:** 2016-11-18

**Authors:** Yusheng Li, Wenfeng Xiao, Ping Wu, Zhenhan Deng, Chao Zeng, Hui Li, Tuo Yang, Guanghua Lei

**Affiliations:** 1 Department of Orthopaedics, Xiangya Hospital, Central South University, Changsha, Hunan Province China; 2 Department of Emergency Medicine, Xiangya Hospital, Central South University, Changsha, Hunan Province China

**Keywords:** Osteoarthritis, Knee, SIRT1, Articular cartilage, Improved Mankin scoring

## Abstract

**Background:**

The study aims to investigate the expression of SIRT1 in articular cartilage of patients with primary knee osteoarthritis (OA) and its relationship with disease severity.

**Methods:**

Cartilage tissue samples were collected from 38 knee OA patients and 9 normal healthy controls and then ascribed to normal, mild, moderate, and severe groups on the basis of the improved Mankin grading system. The expression of SIRT1 in articular cartilage was detected by immunohistochemistry and western blots. The expression of p53 and acetylated p53 (Ac-p53) was also measured by western blots.

**Results:**

The mutual comparisons of the SIRT1 expression levels in all groups have statistical significance except the one between the mild and moderate groups. Moreover, western blot results showed that the SIRT1 was decreased and p53/Ac-p53 were increased in the OA group. The average gray level of SIRT1 increases with the improving grade of the improved Mankin grading system scorers.

**Conclusions:**

The expression of SIRT1 in articular cartilage is negatively associated with severity of knee OA, indicating that SIRT1 may act as a monitoring indicator for determining development and progression of knee OA.

## Background

Osteoarthritis (OA) is a chronic degenerative joint disease resulting in substantial morbidity, physical disability, and reduced quality of life [1]. OA affects up to 15% of the adult population and represents the second greatest cause of disability worldwide, with a huge impact on society both in terms of quality of life for the individuals and high costs for the government [2]. Exploring the gene products that prolong aging in humans as well as detecting mechanisms underlying chondrocyte apoptosis is of great value, for the reason that OA is an age-related disease, the pathogenesis of which may include a variety of biochemical and biomechanical factors [3–5].

SIRT1, a member of the silent information regulator 2 family, is a class III protein deacetylase. SIRT1 has been confirmed to enhance cell survival and inhibit apoptosis through regulating several transcription factors, including p53, the transcriptional coactivator p300, the DNA repair factor Ku70, forkhead box protein O, and nuclear factor-κB (NF-κB) [6–10]. SIRT1 targets are involved in essential biological processes including stress responses, DNA repair, and inflammation, which are important factors in aging and age-related disease [11, 12]. The ability to promote chondrocyte survival and affect chondrocyte differentiation and proliferation has been proved by previous studies, indicating that SIRT1 can protect cartilage degeneration via inhibiting apoptosis and elevating cartilage-specific gene expression [13–15].

However, as far as we know, there have been no detailed studies investigating SIRT1 expression levels in articular cartilage of patients with various stages of OA of the knee. We hypothesized that SIRT1 in articular cartilage may be associated with disease severity in patients with OA. Therefore, the study reported herein was to investigate SIRT1 expression in the articular cartilage of patients with primary OA of the knee and identify the possible correlations with the modified Mankin score of OA, which may serve as a useful tool for indicating the disease severity and progression of OA of the knee.

## Methods

### Patients and samples

This study was approved by the medical ethics committee of Xiangya Hospital, Central South University (grant number 201212063), and all patients had signed informed consent. According to the criteria of the American College of Rheumatology [16], 38 participants (aged 52–71) with no history of any form of secondary OA or inflammatory joint diseases, including rheumatoid arthritis, were eligible for enrollment in this study. The articular cartilage samples were harvested from the destructive area of the tibia plateau when the patients were undergoing total knee arthroplasty. Control experiments were carried out on nine normal cartilage samples which were collected from nine individuals who had suffered above knee amputation due to severe trauma. The patients of the control group had no history of secondary OA, knee injury, rheumatoid arthritis, tuberculous arthritis, and OA in other joints and had not received a steroid injection within the previous 3 months. Control subjects were matched with the OA group by age, gender, and body mass index (BMI).

### Sample preconditioning and classification

Biopsies of the cartilage and bone were obtained from the lateral and medial sides of the tibia plateau, including the loading zone and the margin zone whenever possible [17]. Then, samples containing a cartilage surface approximately 2.0 × 0.5 cm were then fixed in freshly prepared 4% paraformaldehyde, decalcified in diethylpyrocarbonate-treated 0.2 M EDTA (pH 8.0), dehydrated in a grading concentration of ethanol and xylene, and embedded in paraffin. Serial sections of 5 μm were collected for H&E and safranin-O/fast green staining. After that, all samples were divided into four groups, namely, normal, mild, moderate, and severe groups, according to osteoarthritic change levels measured by the improved Mankin grading system [18] as follows: Mankin score 0, normal cartilage with a smooth surface and a regular zonal distribution of chondrocytes; Mankin score 1–4, cartilage surface shows fibrillations and a superficial loss of proteoglycans (safranin-O staining), but the zonal structure is intact; Mankin score 5–8, cartilage samples have clefts reaching down to the middle cartilage zone, and clusters of proliferating chondrocytes are present; and Mankin score ≥9, severely affected cartilage samples with clefts reaching down to the deep zone, in which the tangential zone is lost and chondrocyte clusters are present.

### Immunohistochemistry

Serial sections were deparaffinized and rehydrated before quenching of endogenous peroxidase activity with 3% H_2_O_2_ in methanol for 30 min at room temperature. The cover slips were incubated in 5% bovine serum albumin buffered with phosphate-buffered saline (PBS) solution for 30 min in order to block the nonspecific antibody binding, incubated with rabbit anti-human SIRT1 antibody (E104, 1:40; Abcam, Cambridge, UK) at 37 °C for 2 h, then incubated with biotinylated goat anti-mouse IgG and SABC complex at 37 °C for 30 min after another rinse. Finally, the samples were stained with diaminobenzidine tetrachloride (DAB) and the cover slips were counterstained with hematoxylin. The combined clips and cover were dehydrated with a non-aqueous mounting medium. Meanwhile, a negative control was prepared using the same procedure without primary antibody incubation.

A microscope (high-magnification, Olympus Corporation, Tokyo, Japan) was used for SIRT1 expression evaluation. Blind method was applied for histological assessment by a skilled pathologist. Positive SIRT1 immunostaining was defined as detectable immunoreactivity in the perinuclear or other cytoplasmic regions in the chondrocytes. Average gray values were used for visualization and quantification of relative SIRT1 distribution and expression level. Scanned autoradiograms with medical image analysis software (MIAS)-4400 and ImageJ software were used for semi-quantitative assessment of mean average gray values of SIRT1 expression, as described before [19]. Analysis was defined to a site from surface to the cartilage-bone junction, and grayscale images were taken and converted to absorbance units. PBS solution was used for density standardization and the experiment was repeated thrice. Means of three sections per sample were recorded to minimize the error caused by small variation in section thickness. The coefficient of variation of SIRT1 expression in the cartilage tissues was <2%.

### Western blots

The tissue samples were collected and sonic with a lysis buffer (Beyotime Biotech, Beijing, China) containing 1 mM PMSF. The lysates were centrifuged and supernatants were subjected to western blot analysis. The protein concentrations were measured using the BCA method. The 20–50 μg of protein samples was subjected to 10% SDS-PAGE and then transferred onto PVDF membranes. After blocking with 5% non-fat milk, the membrane was probed with anti-SIRT1, anti-p53, and anti-acetylated (Lys382) antibodies and further probed with secondary antibodies (Santa Cruz Biotechnology, Dallas, TX, USA). Proteins were visualized with an ECL kit (Thermo Fisher Scientific, Waltham, MA, USA).

### Statistical analysis

Ten randomly selected regions of the SIRT1 immunohistochemical staining slices were applied for average gray value measurement by static gray analysis using the MIAS. SPSS 16.0 (version 15.0 for Windows; SPSS Inc., Chicago, IL, USA) was used for data management and statistical analysis. Data were expressed as the mean ± SD. One-way analysis of variance was conducted to determine the differences in the mean values between multiple groups. Spearman’s correlation and linear regression were employed to examine the correlations between the average gray values of SIRT1 expression and the improved Mankin scoring. Differences with *P* < 0.05 were considered as statistically significant.

## Results

### Improved Mankin scoring system in each group

Forty-seven biopsies were obtained from the participants then divided respectively into four groups (normal, mild, moderate, and severe), based on the improved Mankin scoring system. Of the included samples, 9 were classified as the normal group (Mankin score 0), 10 were determined to the mild group (Mankin score 1–4), 13 were classified as the moderate group (Mankin score 5–8), and 15 were classified into the severe group (Mankin score ≥9) (Table 1).

**Table 1:** Improved Mankin scoring system of cartilage in the OA and control groups (mean ± SD)

Group	Sample (*n*)	Mankin score
Normal	9	0.44 ± 0.53
Mild OA	10	3.70 ± 1.06
Moderate OA	13	7.54 ± 1.05
Severe OA	15	12.20 ± 1.37

### Decreased expression of SIRT1 in OA cartilage tissues

**Fig. 1:**
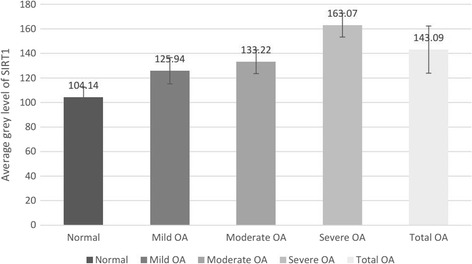
Histogram of SIRT1 average gray level in the OA and control groups

Both the normal and OA groups detected SIRT1 expression by immunohistochemistry, the expression levels of which were compared in the various degrees of degenerated cartilage samples (Fig. 1). The OA group exhibited lower articular cartilage SIRT1 expression levels compared to the healthy controls (143.09 ± 19.23 versus 104.14 ± 8.30, *P* < 0.05). Likewise, the severe group had lower SIRT1 expression levels, namely, higher average gray values, compared to the moderate, minor, and normal groups (163.07 ± 9.78 versus 133.22 ± 9.62, 125.94 ± 10.70, and 104.14 ± 8.30, respectively; Table 2). The mutual comparisons between these groups were demonstrated to be statistically significant (*P* < 0.05), with the exception of that between the mild and moderate groups (*P* > 0.05), suggesting that expression of SIRT1 was dramatically decreased in the impaired cartilage.

**Table 2:** Average gray level of SIRT1 in the OA and control groups (mean ± SD)

Group	Sample (*n*)	Average gray level
Normal	9	104.14 ± 8.30
OA		
Mild	10	125.94 ± 10.71^a^
Moderate	13	133.22 ± 9.62^a,b^
Severe	15	163.07 ± 9.78^a,c,d^
Total	38	143.09 ± 19.23^a^

^a^ *P* < 0.05, versus normal group

^b^ *P* > 0.05, versus mild group

^c^ *P* < 0.05, versus mild group

^d^ *P* < 0.05, versus moderate group

**Fig. 2:**
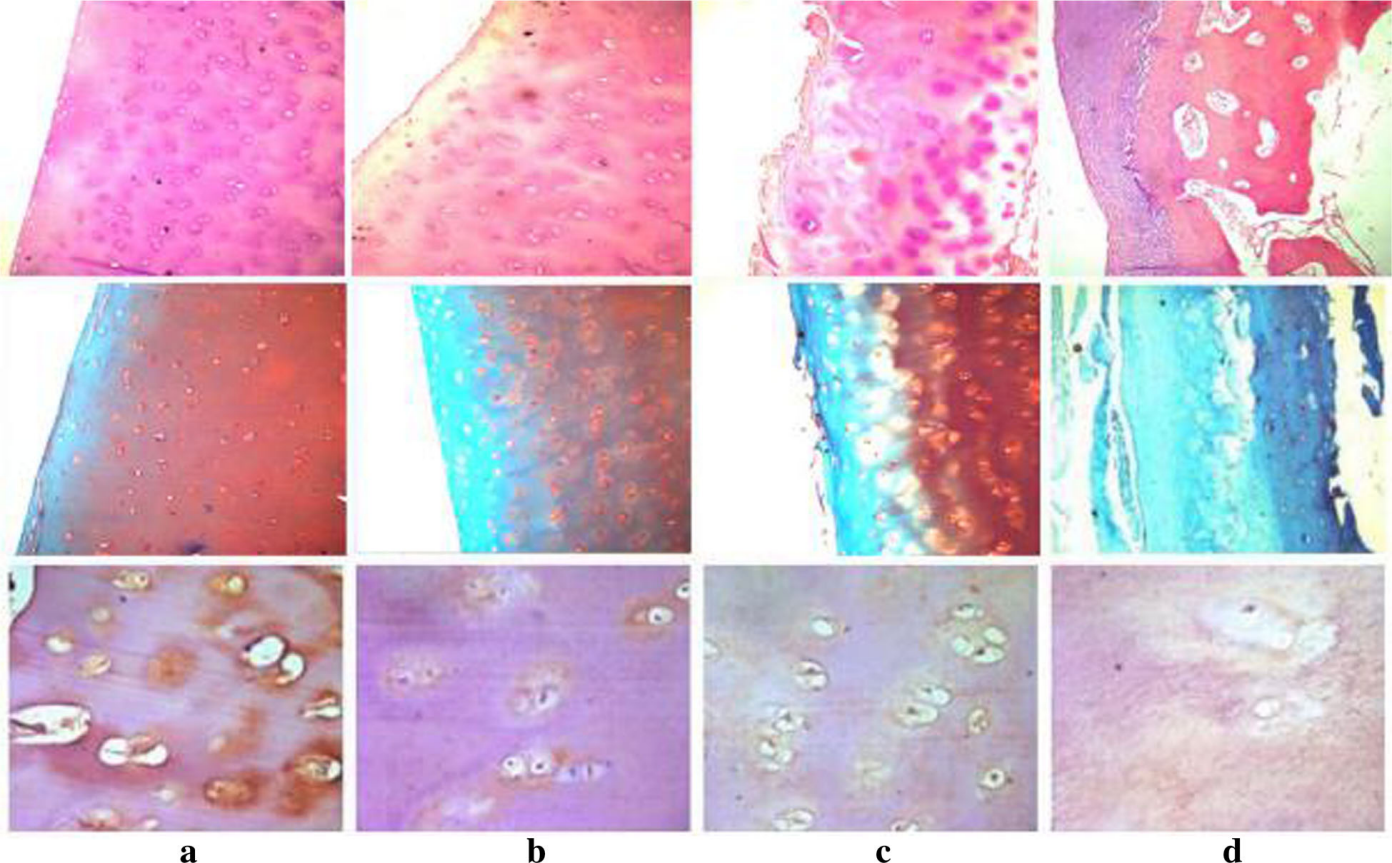
The upper image was visualized with H&E staining of the cartilage (×100), the middle image was visualized with safranin-O-fast green staining (×100), and the lower image was visualized with SIRT1 immunohistochemical staining (×400). **a** Normal group. **b** Mild OA group. **c** Moderate OA group. **d** Severe OA group

H&E staining reveals normal cartilage with a smooth surface and a regular zonal distribution of the chondrocytes. The normal safranin-O-/fast green and SIRT1 staining results are depicted in Fig. 2a, revealing a slightly rougher cartilage surface, irregular chondrocyte arrangement, and several chondrocyte clusters. The mild or moderate safranin-O/fast green and SIRT1 staining in the mild OA group (Fig. 2b) show fibrillations in the cartilage surface and clefts reaching down to the middle cartilage zone, along with the presence of clusters of proliferating chondrocytes. Deep safranin-O/fast green and SIRT1 staining in the moderate OA group (Fig. 2c) reveal obvious chondrocyte loss, with the cartilage surface exhibiting broader fibrillations and clefts reaching down to the deep zone and a loss of the tangential zone. Finally, the loss in the safranin-O/fast green and SIRT1 staining results in the severe OA group can be seen in Fig. 2d.

**Fig. 3:**
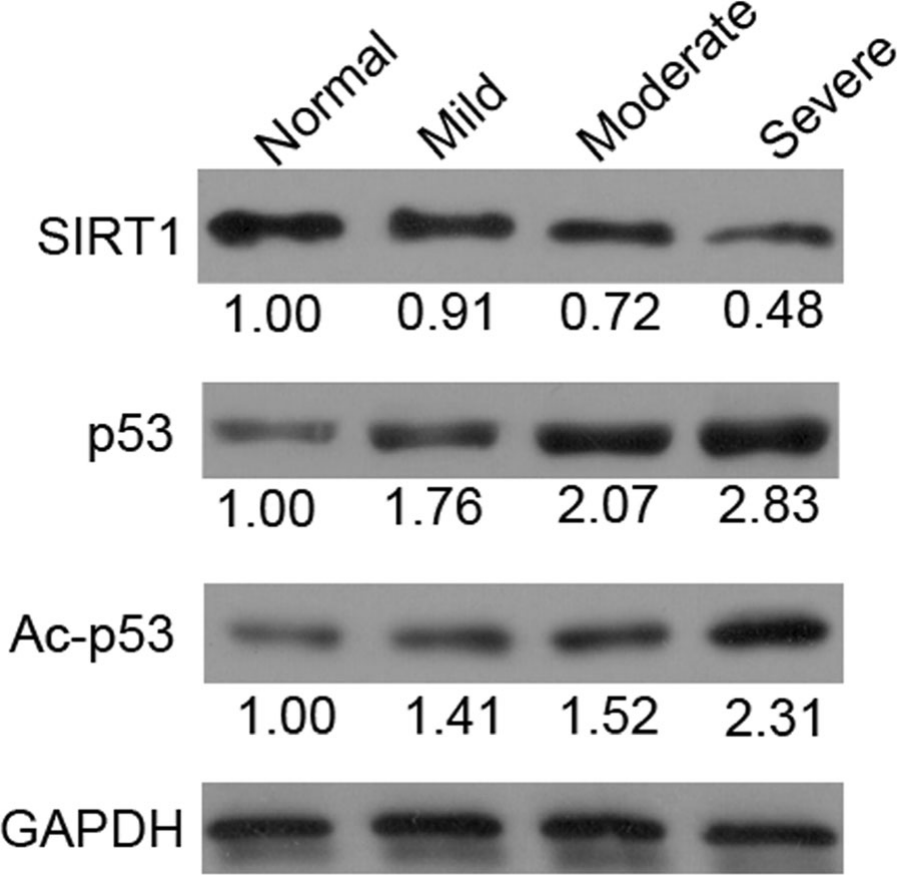
The protein expressions of SIRT1, p53, and acetylated p53 (Ac-p53) were detected by western blot

Moreover, we also used western blots to measure the expression of SIRT1 and its downstream gene p53 in the normal and OA groups (Fig. 3). Similar to the immunohistochemistry results, the expression of SIRT1 was decreased significantly with the improving OA severity. In the contrast, the p53 expression and its acetylation level were dramatically increased in the OA groups, which were positively related to OA severity.

### Correlations between SIRT1 expression and improved Mankin scoring

**Fig. 4:**
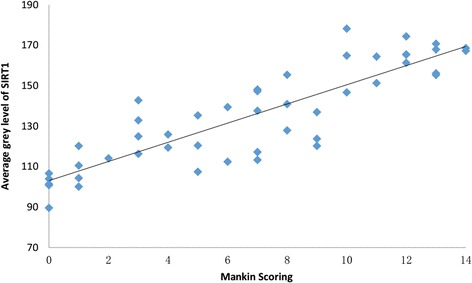
Correlation between the average gray value of SIRT and Mankin scoring. Pearson’s correlation coefficient, *r* = 0.893 (*P* < 0.01)

To reveal the relationship between SIRT1 expression and improved Mankin scoring in OA cartilages, SPSS software was used for assessment of the average gray value of SIRT1 expression in each sample. Pearson’s correlation coefficient was used to determine the correlation between the average gray value of SIRT1 expression and improved Mankin scoring (*r* = 0.893, *P* < 0.01; see Fig. 4). Thus, the expression of SIRT1 correlated with the degenerate level of cartilage negatively, namely, the expression of SIRT1 decreases in accordance with OA cartilage degradation.

## Discussion

SIRT1, a member of the histone deacetylases family, is related to multiple age-associated diseases on the account of its capacity to deacetylate histones and non-histone proteins. SIRT1 expression is clearly detected in healthy cartilages but rarely observed in OA samples [20]. This study found a marked decrease in SIRT1 expression levels in articular cartilage of patients with knee OA in comparison with normal controls.

Our observations are consistent with previous findings that SIRT1 is highly expressed in the less damaged and normal human articular cartilage, while it is decreased in severely degenerated cartilage [21, 22]. Dvir-Ginzberg demonstrated that increased protein levels or activity of SIRT1 is responsible for a significant elevation of cartilage-specific gene expression in chondrocytes derived from patients with OA, while decreased protein levels or activity of SIRT1 led to weaken such gene expression [21]. Fujita demonstrated that the similar phenomenon in cartilage samples of OA:SIRT1 expression was barely detected in severely damaged cartilage, whereas high expression of SIRT1 was linked to less degenerated samples. Dramatic OA-like gene expression changes, namely, downregulation of aggrecan and upregulation of COL 10A1 and ADAMT-5, were observed after inhibiting SIRT1 expression via small interfering RNA (siRNA). This study revealed that SIRT1 could slow down the disease progression in chondrocytes and its deficiency may lead to chondrocyte hypertrophy and cartilage matrix loss and ultimately result in OA [22]. Furthermore, Matsuzaki identified that SIRT1 protein first increased in the early phase of OA development and then gradually decreased in the advanced OA stage in cartilage-specific SIRT1-conditional knockout mice models of OA [23]. These findings suggest that SIRT1 may defend against cartilage degeneration during the development and progression of OA.

The most eminent change of OA is cellular matrix damage and the loss of tissue cellularity [24]. Apoptosis, also called programmed cell death, has been considered to play a role in the OA process [4, 5]. SIRT1 expression in chondrocytes has an anti-apoptotic effect involving various pathways [13, 15, 20, 25]. The p53 protein is one of the first deacetylated non-histone proteins reported to interact with SIRT1 [6] . Under DNA damage and other cellular stress, p53 protein levels are markedly upregulated and their activities are induced by phosphorylation and acetylation. The acetylation of p53 is indispensable for apoptosis and inhibition of cell growth [26]. Thus, deacetylation by SIRT1 modulates the function of p53. In this study, along with the improving OA severity, SIRT1 was decreased. Consequently, p53 and Ac-p53 were both dramatically increased in the OA groups, indicating occurrence of apoptosis in OA tissue. To study the effect of SIRT1 on apoptosis, Takayama inhibited SIRT1 by siRNA and activated it using resveratrol during nitric oxide (NO)-induced apoptosis. Expression of cleaved caspases as well as Bax and Bcl-2, mitochondria-related apoptotic signaling proteins, was detected in the mitochondrial fraction. SIRT1 causes Bax level elevation and reduction, while resveratrol functions in the opposite way, which indicates that SIRT1 could regulate apoptosis in chondrocytes through modulating mitochondria-related apoptotic signals [15]. Cell survival of chondrocytes was regulated by SIRT1 via the downregulating protein tyrosine phosphatase 1B (PTP1B), which is a kind of effective chondrocyte pro-apoptotic protein highly expressed in osteoarthritic cartilage [13]. As a cytokine that mediates joint inflammation in arthritis, tumor necrosis factor α (TNF-α) was also found able to decrease SIRT1 activity and induce its cathepsin B-mediated cleavage and result in cartilage-specific gene expression inhibition in TNF-α-treated cells [25]. Oppenheimer also proved that SIRT1 could block TNF-α-induced apoptosis in chondrocytes from OA patients following exposure to pro-inflammatory cytokines [20]. When comparing musculoskeletal features, OA severity, and chondrocyte apoptosis in articular cartilage between heterozygous haploinsufficient (SIRT1 (+/−)) and wild-type (WT; SIRT1 (+/+)) 129/J mice, Gabay observed increased apoptotic chondrocytes and advanced OA progression in heterozygous SIRT1 knockout (CKO) mice [27]. Together, these observations suggest that SIRT1 plays a preventive role in the apoptotic process and that accelerated OA progression in SIRT1-CKO mice might be caused in part by increased apoptosis of chondrocytes [23].

Limitations of the study should be acknowledged. Firstly, the study had a small sample size and was a single-center project. A more definitive conclusion may be drawn if larger scale, multi-center investigations are carried out. Secondly, the study suffered potential selection bias, as our sample comprised only patients with knee OA, attending Xiangya Hospital in China’s Central South University. Thirdly, the study had a cross-sectional design, and therefore, no conclusions with regard to cause-and-effect relationships can be drawn. Finally, cartilage from severe trauma patients was used as the control sample, and these patients cannot be considered the exact equivalent of healthy individuals because we lack evidence confirming that trauma and related complications do not affect SIRT1 concentrations in cartilage.

## Conclusions

This study detected down-expression of SIRT1 in articular cartilage of patients with knee OA, and the expression levels are negatively associated with disease severity, indicating that SIRT1 may induce pathogenesis and progression of knee OA. SIRT1 may serve as a monitoring indicator for determining the development of knee OA. However, further studies are in progress to elucidate the contribution of SIRT1 to the pathogenesis of the degenerative process of OA.

## Abbreviations

CKO: Conditional knockout
DAB: Diaminobenzidine tetrachloride
MIAS: Medical image analysis software
NF-κB: Nuclear factor-κB
NO: Nitric oxide
OA: Osteoarthritis
PBS: Phosphate-buffered saline
PTP1B: Protein tyrosine phosphatase 1B
siRNA: Small interfering RNA
TNF-α: Tumor necrosis factor α
